# A Pressure-Pad-Embedded Treadmill Yields Time-Dependent Errors in Estimating Ground Reaction Force during Walking

**DOI:** 10.3390/s21165511

**Published:** 2021-08-17

**Authors:** Prabhat Pathak, Jooeun Ahn

**Affiliations:** 1 Department of Physical Education, Seoul National University, Seoul 08826, Korea; prabhat@snu.ac.kr; 2 Institute of Sport Science, Seoul National University, Seoul 08826, Korea

**Keywords:** vertical ground reaction force (VGRF), Zebris treadmill, capacitive sensors, walking, measurement reliability, estimation errors, curve-fitting model

## Abstract

Accurate and reliable vertical ground reaction force (VGRF) measurement is essential in various biomechanical and clinical studies. Recently, pressure–pad-embedded treadmills have been widely used for VGRF measurement as a relatively less expensive option than the force platform-mounted treadmills. Prior studies have shown that the popular Zebris treadmill is reliable when used to measure peak VGRF for short walking sessions. However, comprehensive evaluation of human walking requires information of gait parameters over sufficient gait cycles. In this study, we quantify the long-term temporal changes in VGRF values measured by the Zebris treadmill. Twenty participants walked on the treadmill for 10 min twice, with 10 min rest between trials. We found an evident decline in the measured VGRF and impulse over time for both trials. The Zebris system also consistently yielded the lower VGRF values during the second trials. These results indicate that the Zebris treadmill is unreliable in measuring VGRF during walking, and a 10 min break is not enough for the embedded sensors to recover their sensitivity. We provided a way to resolve these time-dependent errors; using the impulse-momentum theorem and collected kinematics of the participants, we formulated a curve-fitting model encapsulating the growing VGRF estimation error.

## 1. Introduction

The vertical ground reaction force (VGRF) is the major component of a salient external force acting on the body during locomotion. Accurate and reliable measurement of VGRF is necessary for estimating the precise load exerted on the body segments and joints [1,2,3,4]. VGRF has been typically measured using a force platform fitted with load cells or force transducers mounted on the ground or a motorized treadmill. Numerous studies have shown that the measurement of VGRF using these types of force platforms is highly reliable [5,6,7,8]. However, continuous VGRF measurement using floor-mounted force platforms is difficult, and force plate-mounted motorized treadmills are costly. Relatively less expensive pressure-pad-embedded motorized treadmills are developed to overcome these drawbacks. Among numerous brands, capacitive pressure sensor-embedded Zebris treadmills are extensively used to measure spatiotemporal and kinetic gait parameters. Several studies have shown high reliability of Zebris treadmills for measuring spatiotemporal gait parameters [9,10,11,12]. However, studies verifying the reliability of such a system for measuring VGRF are limited.

Technologies that use capacitive sensors to measure plantar pressure show drift during VGRF measurement [13,14,15]. Common reasons behind the drift are temperature changes, hysteresis, and prolonged pressure-induced sensor deformation [15,16]. Several studies have assessed the reliability of such capacitive sensor-based systems, including Zebris treadmills. Item-Glatthorn et al. reported highly reliable peak forefoot and heel forces measured between-days when participants walked on a Zebris treadmill for 20 s with increasing speeds and inclinations [17]. In another study, Nüesch et al. reported highly reliable peak forefoot and heel forces measured between-trials and days when participants walked and ran on a Zebris treadmill for 2 min with increasing speeds [10]. In contrast, Van Alsenoy et al. reported only moderate reliability of peak VGRF measured between-trials and days when participants ran on a Zebris treadmill for 20 s with increasing speeds [18]. In addition, the peak VGRF values measured during the second trial were lower than the values during the first trial for all running speeds; the peak VGRF was underestimated in the subsequent trials. Considering that reliable assessment of gait stability and variability requires a sufficient number of gait cycles [19,20,21,22], these previous studies that addressed the reliability of VGRF measurement using a Zebris treadmill have an important drawback; the gait parameters were recorded only for a short period of time. Furthermore, the previous studies only used one single peak force during walking or running to check the reliability of VGRF measurement despite the fact that VGRF keeps changing with time.

In addition, the capacitance-based pressure sensors embedded into the Zebris treadmill have a spatial resolution of 8.5 mm [18,23], whereas the international guideline for the maximum spatial resolution of the pressure sensor for an accurate measure of plantar force is 5 mm [24]. Any system with a coarse spatial resolution plausibly underestimates the maximum plantar pressure because the total force is to be divided by a larger area [25,26]. Using experimental data, Lord modeled plantar pressure distribution during standing and found 67% accuracy when plantar pressure was measured using sensors with a spatial resolution of 10 mm [27]. Pataky estimated the accuracy for sensors with various values of the spatial resolution using the model proposed by Lord and standing plantar pressure data measured by Xsensor and Zebris pressure pads [28]. In contrast with the results from Lord’s study, Pataky found 90% accuracy for sensors with spatial resolution between 1.7 mm and 17.4 mm. However, he postulated that changes in the foot positioning could have non-trivial effects on the plantar pressure and force measurement accuracy.

Unlike standing, walking involves continuous changes in foot positioning. To our knowledge, no mathematical model or systematic method has been proposed to estimate the errors in pressure measurement during walking using a pressure-pad-embedded treadmill. Hence, in this study, we firstly aim to quantify any time-dependent estimation error of the Zebris treadmill when the device is used to measure the VGRF of walking for a prolonged time period. Our results show continuously growing but converging errors in VGRF estimation; Zebris treadmills are unreliable for long-term VGRF measurement during walking. Considering that the researchers in the field of human movement science widely utilize pressure-pad-embedded treadmills to measures VGRF during walking, we secondly aim to develop a viable method to compensate for these errors in VGRF measurement. Using the impulse-momentum theorem, we formulated curve-fitting models encapsulating the temporal declines in the VGRF with high goodness of fit. Our results suggest an effective method for compensating for the low reliability of the Zebris treadmill and similar capacitive pressure sensor-embedded treadmills for measuring VGRF during walking.

## 2. Materials and Methods

### 2.1. Participants

We recruited 20 healthy young adults (16 men and 4 women; age: 28 ± 4.68 years; height: 1.73 ± 0.08 m; weight: 72.10 ± 12.64 kg). This number of the participants is larger than the sample size required for a statistical power of 0.95. We conservatively set the effect size between small and medium values as 0.35 [29]. We then selected the expected power and p-value for statistical significance as 0.95 and 0.05, respectively. Under these conditions, G-power software calculated the required sample size as 12 [30]. The recruited participants had no known cardiovascular, orthopedic, or neuromuscular disorders. The participants were informed about all the aspects of the study, and they provided informed, written consent before participation. All aspects of the study adhered to the principles and guidelines described in the Declaration of Helsinki and approved by the Institute Review Board of Seoul National University (IRB No. 2007/001-009).

### 2.2. Equipment

We asked the participants to walk on a pressure-pad-embedded treadmill (Model Gait analysis FDM-TDSL-3i, Zebris Inc.^®^, Germany; belt length: 150 cm; belt width: 50 cm; maximum incline: 15%; maximum speed: 24 km/h), which was used to record VGRF at a sampling frequency of 50 Hz. A motion capture system consisting of ten infra-red cameras (Optitrack Prime^X^ 13, Natural Point, Inc., OR, USA) was used to record coordinates of the retro-reflective markers attached to the participant at a sampling frequency of 100 Hz. A sync box was used to synchronize the initiation of data acquisition from the two systems.

### 2.3. Experimental Procedure

Initially, we estimated the preferred walking speed (PWS) of the participants. To measure PWS, we asked each participant to walk on the treadmill, starting at a speed of 2.5 km/h. We then increased the speed by 0.1 km/h every 10 s and asked the participants to report once they perceived the speed that best described their normal walking speed. We further increased the speed by 1.0 km/h and decreased it by 0.1 km/h, again asking the participants to report once they perceived the speed that best described their normal walking speed. We repeated this process three times and used the average speed as PWS. We measured PWS within one week prior to the main experiment.

We attached 36 retro-reflective markers on the anatomical landmarks of the head: top, left, right, and front temple; and left and right sides of the upper and lower body: acromion, medial and lateral epicondyle of the humerus, radius- and ulna-styloid process, third metacarpal, posterior superior iliac spinae, anterior superior iliac spinae, greater trochanter, medial and lateral epicondyle, medial and lateral malleolus, first and fifth metatarsal, and heel. After attaching the retro-reflective markers, we measured the participant’s body weight (BW). We then asked the participants to perform two walking trials, 10 min each, on the treadmill at their PWS. Each 10 min trial consisted of two 5 min data acquisition sessions because the Zebris treadmill can record data only up to 5 min for a single session. It took 5 to 10 s to save the file for the first session and start the second data acquisition session. Participants were given 10 min to rest between trials. We also measured the participant’s BW between trials and right after the final trial to check for any possible change in the BW.

### 2.4. Data Processing

**Figure 1:**
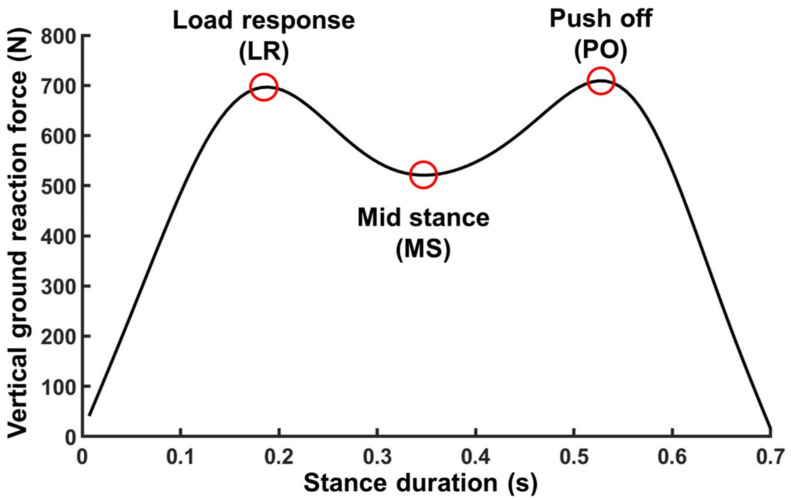
An illustration of the VGRF curve for a single step. The red circles denote three stereotypical time points for each step; the time point of load response (LR), mid stance (MS), and push off (PO). The LR and PO are the time points of two peaks of the double bump pattern of the VGRF curve, and MS is the time point of the minimal value of VGRF between LR and PO.

The VGRF curve was extracted for each step during the 10 min walking trials, separately for the left and right foot. We then extracted the specific VGRF values at three stereotypical time points for each step; the time point of load response (LR), mid stance (MS), and push off (PO). We selected LR and PO as the time points of two peaks of the double bump pattern of the VGRF curve and MS as the time point for the minimal value of VGRF between LR and PO (Figure 1). In addition, we calculated impulse by integrating VGRF over time. We also calculated the stance duration of each step (the elapsed time between initial contact and toe-off) and the stride duration. The VGRF values at each of the three time points, impulse, stance duration, and stride duration were separately averaged over every one-minute interval. 

We filtered the raw coordinates of the retro-reflective markers using a zero-lag, fourth order low pass filter with a cut-off frequency of 10 Hz. Using biomechanical modeling software, Visual 3D (Visual3D v6^TM^, C-Motion, Inc., Germantown, MD, USA), we built a fifteen-segment model, which included left and right foot, shank, thigh, upper arm, forearm, and hand, trunk, and head segments. We identified the whole-body center of mass (COM) using this model, and calculated the vertical velocity of COM (*V*_COM_).

### 2.5. Statistical Analysis

We conducted a two-way repeated measures analysis of variance (ANOVA) to evaluate any statistical differences between the VGRFs at LR, MS, and PO; impulses; stride durations; stance durations; and changes in the vertical velocities of COM for 20 participants depending on time (10 levels: 1 to 10 min) and trial (two levels: trials 1 and 2) separately for the left and right foot. We performed Mauchly’s sphericity test to evaluate the assumption of sphericity. If the assumption of sphericity was violated, the Greenhouse-Geisser criterion was used to reduce the degrees of freedom. Bonferroni correction was used as a post-hoc test for multiple pairwise comparisons over time and between trials. The level of statistical significance was set at *p* < 0.05. Additional statistical analysis methods used to evaluate the goodness of fit of the curve-fitting models to encapsulate the errors in the VGRF estimation are clarified in Section 5.

## 3. Results

**Figure 2:**
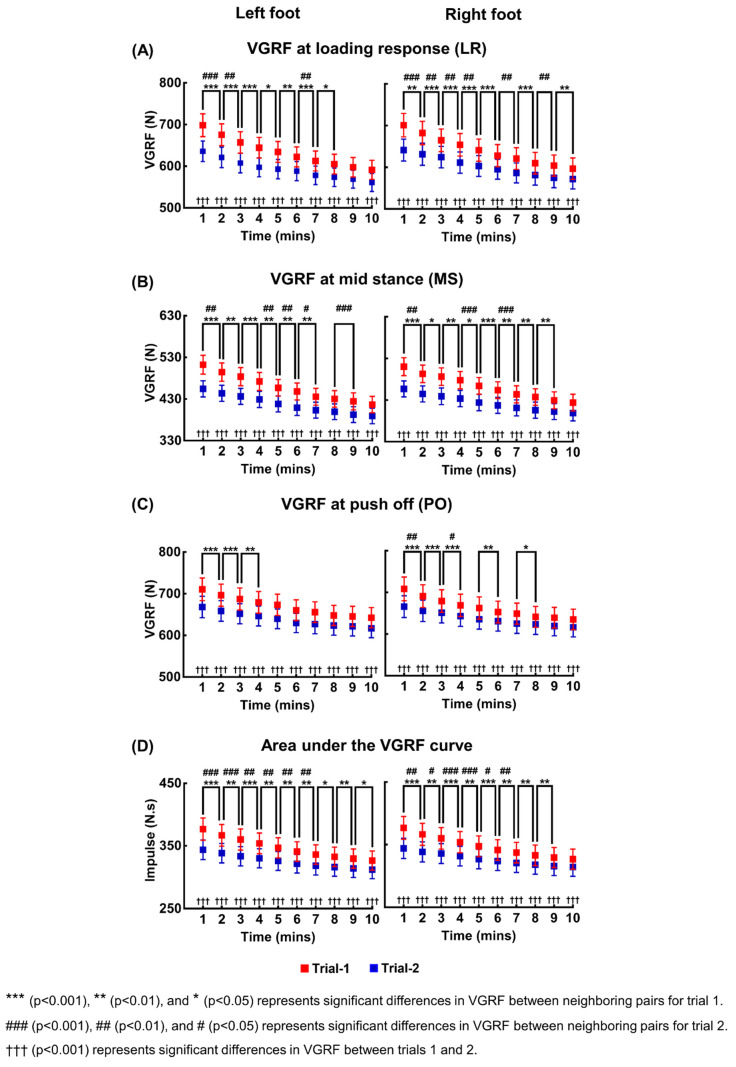
Changes in the VGRFs at the three stereotypical time points and the impulse due to VGRF with increase in time. (**A**–**C**) show the means and standard error bars of VGRFs for 20 participants at LR, MS, and PO, respectively, averaged over every one-minute time interval for the both feet and two trials. (**D**) shows the means and standard error bars of impulse for 20 participants averaged over every one-minute time interval for the both feet and two trials.

The means and standard errors of VGRFs at LR, MS, and PO, and the impulse due to VGRF of 20 participants, are shown in Figure 2. For all four measures (VGRFs at three time points and the impulse), two-way repeated measures ANOVA showed significant main effects of time and trial, and significant interaction between time and trial for both feet. The statistical results are summarized in Table 1.

For both trials and both feet, pairwise comparisons revealed that the estimated VGRFs and impulse during a time interval of one minute were less than the estimated values during the next minute with statistical significance in most neighboring pairs. The number of neighboring pairs without statistically significant change always increased in the second trial compared to the first trial for all the four measures and both feet. Pairwise comparisons between trials also revealed that the VGRFs and impulse of 20 participants in the first trial were significantly larger than those in the second trial at any one-minute time interval for both feet.

**Figure 3:**
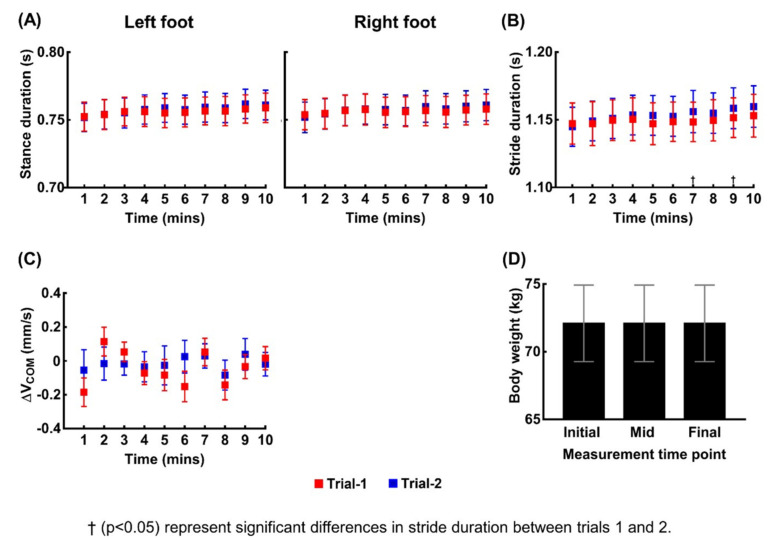
The measures used to assess whether the time-dependent decrease in the VGRF is due to alterations in kinematics or dynamics during walking. (**A**–**C**) show the means and standard error bars of the stance durations of the both feet, stride durations, the difference in the values of the vertical center of mass velocity between the beginning of one stride and the next one (Δ*V*_COM_) for 20 participants averaged over every one-minute time interval for the two trials. (**D**) shows the means and standard error bars of the body weights (BW) for 20 participants for three measurement time points: initial (before the first trial), mid (between the first and second trial), and final (after completion of the second trial). BW remained exactly same for all participants across the three time points.

We confirmed that the temporal decline in the estimated VGRFs and impulses are due to imperfect sensing from the pressure pad rather than changes in walking kinematics and dynamics by assessing four measures: (1) BW, (2) stance duration, (3) stride duration, and (4) ΔV_COM_ (the difference between the V_COM_ values at the beginning of one stride and the next one). The mean and SE of these four measures of 20 participants are shown in Figure 3, and the results of the statistical analysis are summarized in Table 2. In contrast with the ANOVA results for VGRF and impulse, two-way repeated measures ANOVA concluded no significant main effect except for the effect of time on the stance duration of the left foot. Even for the only observed effect of time on the stance duration of the left foot, the post-hoc pairwise comparisons concluded no significant difference over each one-minute interval. In addition, BW remained exactly the same for all participants across the three BW measurement time points. Therefore, stance duration, stride duration, BW, and ΔV_COM_ were seldom affected either by walking duration or the trial number.

## 4. A Curve Fitting Model of the Estimation Error

According to the impulse-momentum theorem, the time integration of the net vertical force applied to the participant should be identical to the change in the vertical linear momentum of the participant, i.e.,

(1) $$I=\int \left(VGRF_{total}-mg\right)dt=m∆V_{COM}\;,$$

where the interval of integration is from a right heel strike to the subsequent right heel strike; *VGRF_total_* is the total VGRF on the left and right foot; *m* is the mass of the participant; and *g* is the gravitational acceleration. In typical walking, the kinematics is almost periodic, and therefore *V*_COM_ after one gait cycle is expected to remain similar to the value at the beginning of the cycle. Experimental data agree with this expectation; *V*_COM_ remains almost constant, making Δ*V*_COM_ close to zero throughout 10 min of walking (Figure 3C). However, experimental results also showed a time-dependent decline in VGRF, whereas BW remains almost constant. Hence, the left hand side of Equation (1) should decrease, whereas the right hand side remains close to zero. This discrepancy clarifies the time-dependent decline in the estimated impulse. Combined with the time-dependent decline in VGRFs and almost constant stance durations (Figure 3A), the experimental results conclude that the embedded pad underestimates the pressure, and the error grows as the operation time of the treadmill increases, i.e., if we calculate the error in the measured impulse as the difference between the impulse estimated from the Zebris system, which is $\int \left(VGRF_{total}-mg\right)dt$, and the actual impulse obtained from kinematics, *m*Δ*V*_COM_, then, the magnitude of the error (Δ*I*) will grow as time, stride number, or the traveled distance increases.

We formulated a model encapsulating the evolving error. After inspecting the general shape of the curves in Figure 2, we assumed that exponential decay would be an appropriate approximation. Therefore, we fit the dynamics of the estimation error as,

(2) $$\Delta I_{fit}=\Delta I_{steady}+\left(\Delta I_{initial}-\Delta I_{steady}\right)e^{-k\left(x-x_{initial}\right)},$$

where Δ*I_fit_* is the fitted value of the error in the impulse measurement; Δ*I_steady_* is the steady-state value which Δ*I_fit_* will approach after sufficiently many strides; *k* is the exponential decay constant; *x* is the stride number; and *x_initial_* and Δ*I_initial_* are the initial values of the stride and the error, respectively. We set *x_initial_* as one.

To find the optimal Δ*I_initial_*, *k*, and Δ*I_steady_* iteratively, we need to select their proper initial values for the iteration. We chose initial Δ*I_initial_* as the error in the impulse measurement during the first stride. We chose the initial *k* as the overall slope of the impulse error with respect to stride, calculated between the last and first strides. Finally, we chose the initial Δ*I_steady_* as the value of the error at the final stride. We iterated the values of Δ*I_initial_*, *k*, and Δ*I_steady_* using unconstrained multivariate optimization to find the values making Δ*I_fit_* closest to the true error, which we calculated as,

(3) $$\Delta I=\int \left(VGRF_{total}-mg\right)dt-m∆V_{COM}$$

for each stride. The objective function of the optimization was the sum of the squared Euclidean distance between Δ*I* and Δ*I_fit_*, and the maximum number of iterations was set as 1000.

## 5. Goodness of Fit of the Model

We assessed the model’s goodness of fit by calculating the coefficient of determination (*R*^2^) between Δ*I* and Δ*I_fit_* after the iteration, separately for each participant and trial. The values of *R*^2^ indicate the proportion of variance explained by the curve-fitting model used to encapsulate the error between Δ*I* and Δ*I_fit_*. Considering that the model might be sensitive to a slight fluctuation in Δ*I* during each single stride, we additionally assessed the model’s goodness of fit after downsampling the data by averaging Δ*I* over 5, 10, 15, and 20 strides. It is also highly plausible that the error depends more directly on the distance traveled by the treadmill belt or the operation time of the treadmill rather than the stride number. To address this, we calculated the *R*^2^ values after fitting ΔI with respect to time and distance. Again, we performed the curve fitting after downsampling the data by averaging the Δ*I* over 5, 10, 15, and 20 s; and 5, 10, 15, and 20 m.

**Figure 4:**
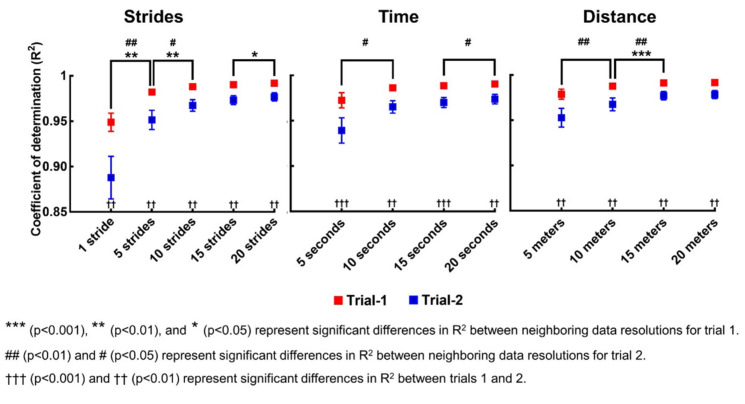
The goodness of fit of the curve fitting model of the VGRF estimation error. The figures from left to right are the means and standard error bars of the coefficient of determination (*R*^2^) for 20 participants when fitting the error (Δ*I*) with respect to the stride number (strides), the operation time of the treadmill (time), and the distance traveled by the treadmill belt (distance), respectively, for the two trials. We assessed the model’s goodness of fit for the curve fitting with respect to every single stride, every 5, 10, 15, and 20 strides. Similarly, we assessed model’s goodness of fit after downsampling Δ*I* by averaging it over 5, 10, 15, and 20 s; and 5, 10, 15, and 20 m for time and distance, respectively. Generally, downsampling the data increased the mean *R*^2^ of 20 participants. The mean *R*^2^ of 20 participants for the first trial was larger than the second trial.

**Figure 5:**
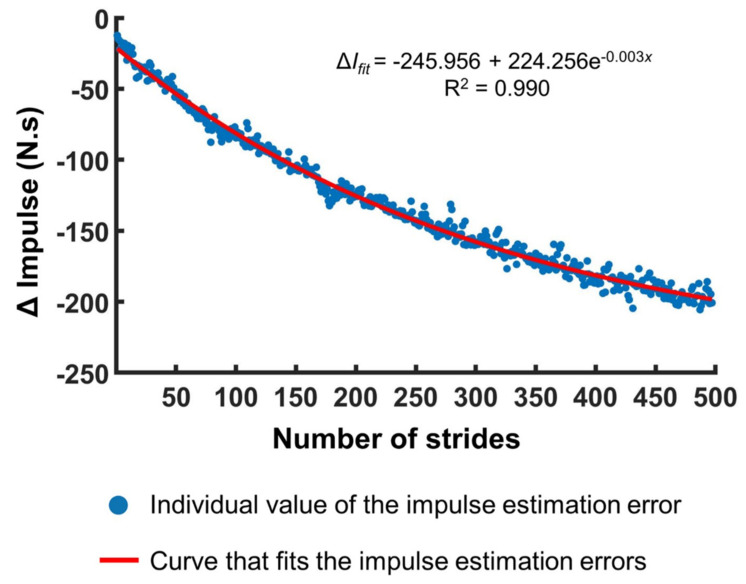
A representative figure illustrating the curve-fitting model that encapsulates the VGRF estimation error over strides. The blue circles are the individual impulse estimation error values for each stride, and the red line is the curve used to encapsulate the temporal changes in the impulse estimation errors.

The means and standard errors of the *R*^2^ values for 20 participants when fitting Δ*I* with respect to strides, time, and distance are shown in Figure 4. The mean *R*^2^ of 20 participants was above 0.88 in every case and above 0.95 in most cases. A representative fitting curve that approximates the growing errors in VGRF estimation is shown in Figure 5. The *R*^2^ values for the first trials were larger than those for the second trial. Downsampling by averaging increased the *R*^2^ values. We performed two-way repeated measures ANOVA to evaluate any statistical differences between the values of *R*^2^ for 20 participants depending on the data resolution (stride: 1, 5, 10, 15, and 20 strides; time: 5, 10, 15, and 20 s; distance: 5, 10, 15, and 20 m) and trial (2 levels: trials 1 and 2). The results are summarized in Table 3. For all three variables (strides, time, and distance), there were significant main effects of data resolution and trial on *R*^2^, and there was a significant interaction between data resolution and trial.

## 6. Discussion

The capacitive pressure sensor-embedded treadmill has been widely used as a reliable alternative to expensive force platform-mounted treadmills. However, the plantar force measured by capacitive sensors inherently shows drift due to its sensitivity to temperature change, hysteresis, and prolonged pressure-induced sensor deformation. When we assessed the VGRF of 20 participants who walked on a capacitive pressure sensor-embedded treadmill, we found a continuous decline in the measured VGRF over time (Figure 1). In addition, when subsequent measurements were performed after 10 min, the latter measurement consistently yielded lower VGRF values compared to the former one.

We rigorously confirmed that the participants’ walking kinematics and bodyweight did not contribute to the decline in the measured VGRF. According to Newton’s 2nd law, the net external force should equal to mass times the acceleration of COM of any system. During walking on a treadmill, the net external force applied to the walker is the sum of the ground reaction force, body weight, and almost negligible air drag; the only remote force applied to the body of the walker is gravitational force or body weight, and the contact forces applied to the walker are only the ground reaction force and air drag. All other forces (e.g., from various muscles) are internal forces, and therefore cannot contribute to the kinematics of the center of mass by virtue of Newton’s 3rd law. Note that the air drag (in the vertical direction) is negligible (or at least almost zero on average per stride) during walking on a treadmill. Therefore, if we integrate Newton’s 2nd law with respect to time, the impulse in the vertical direction $\int \left(VGRF_{total}-mg\right)dt$ should be same as *m*Δ*V*_COM_; the right hand side of Equation (3) should be very close to zero if VGRF, body weight, and Δ*V*_COM_ are all measured correctly. However, this expectation directly contradicts the experimental results. To assess body weight and Δ*V*_COM_, we used the gold-standards: a reliable and accurate scale and a motion capture system. Hence, the only possible explanation for the time-dependent errors is erroneous sensing of VGRF; capacitive sensor-embedded treadmills are not reliable in long-term VGRF measurement during walking. 

In contrast with previous studies that evaluated the reliability of capacitive sensor-embedded treadmills only by assessing peak VGRF values for a short period of time, we quantified relatively long-term time-dependent errors in the measured VGRF. Although this finding has the merit of cautioning researchers to avoid long-term VGRF measurement using the Zebris treadmill or similar systems, we went a step further. We estimated the time-dependent errors using the impulse-momentum theorem and formulated curve-fitting models. It might be possible to model the underlying mechanism of the erroneous measure due to the repetitive mechanical stress that the foot exerts on the capacitive sensors during walking. However, to develop a competent model based on such physical mechanisms, it is necessary to know many parameter values that determine the sensor dynamics, which the treadmill company does not disclose. Further, it is extremely challenging to make an accurate model of the mechanical interaction between the foot and the sensors, and the detailed dynamics inevitably depends on the walking pattern of each participant. Therefore, rather than developing a model based on physical mechanisms, we developed a straightforward curve-fitting model customized to individual participant’s walking dynamics. Iteration of three parameters (rate of decay, initial, and steady-state VGRF estimation errors) enabled the resultant curve to fit the growing errors in VGRF estimation with high *R*^2^ values above 0.95 in most cases (Figure 4). Note that we developed one curve fitting model per each individual participant; the three parameters of the fitting model are different for each participant, which we summarized in the Supplementary Tables S1–S3. Considering that the experimenter should collect the walking data of each participant, deriving a curve-fitting model customized for each participant’s walking dynamics, rather than a general model with universal parameter values, is more adequate and efficient.

Another finding of this study is that the VGRF values measured during the second trials are significantly lower than those measured during the first trials. This finding is consistent with the result of a previous study by Van Alsenoy et al. [18], which reported that the peak VGRF values measured by a Zebris treadmill during the second trial were lower than the values during the first trial for all running speeds. A previous study by Item-Glatthorn et al. reported that a Zebris treadmill provided reliable peak forces measured between-days [17], but the participants walked on a Zebris treadmill only for 20 s in this study. Nüesch et al. also reported that peak forces measured by a Zebris system were consistent across trials and days when participants walked and ran on a Zebris treadmill for 2 min [10]. These studies support that a one-day break may be sufficiently long for the sensors to regain their sensitivity after being used for a short period, such as 20 s or 2 min. However, our result clearly indicates that the underestimation of VGRF becomes more severe as stride number increases during the 10 min of walking in the first trial, and a 10 min break between the trials is not long enough for the sensors to recover from the accumulated effects of mechanisms that cause the underestimation of pressure. It is plausible that the minimum duration of the break session necessary for the pressure pad to regain the original sensitivity depends on the strength of the actual mechanical stress and the time interval during which the sensors were exposed to the stress. This speculation leads to the need for considering the locomotion speed and weight of the participants and the length of the locomotion session to decide the length of the break session that secures a reliable measure of VGRF. Such a concrete design criterion for the experiment should be systematically addressed by additional future work.

In this initial study, we measured VGRF when the participants walked at their PWS, which was within a small range, between 3.1 and 4.2 km/h. For high speed locomotion such as running, VGRF increases substantially [31,32], and therefore may cause further sensor deformation, decreasing the reliability of VGRF measurement. A previous study actually reported underestimation of VGRF by a Zebris treadmill for high speed running [18]. In addition, the magnitude and trend of the measurement errors might depend on the foot strike pattern during running, which can be categorized as fore, mid, or rear foot strike [33,34]. Similarly, patients with foot pathologies or neurological dysfunctions alter pressure distribution and increase the center of pressure trajectory variability during the stance phase [35,36,37,38]. These plantar function irregularities during walking can also skew the time-dependent trend of VGRF estimation errors. Hence, without proper future work, the efficacy of our approach to developing a curve fitting model to encapsulate the VGRF estimation error is not guaranteed for running or pathological gait.

## 7. Conclusions

Measuring foot pressure using capacitive sensors is a cost effective way to obtain VGRF information during walking, but the affordability comes with the cost of inaccuracy. We clarified the time-dependent growing errors in VGRF values measured by a capacitive pressure sensor-embedded treadmill. Our results strongly suggest that researchers should be cautious when using the VGRF values obtained from such systems to evaluate time-dependent locomotor behaviors. As a quick solution to the identified problem, we propose formulating a curve-fitting model customized for each participant. Instead of developing a competent mathematical model of the sensor dynamics and the effect of the repetitive mechanical stress due to foot contact, we suggest simple, quick and straightforward curve fitting based on the obtained data and iteration. This approach may be an effective way to estimate VGRF values during walking more accurately using treadmills with capacitive pressure sensors. Hence, researchers in the field of human movement science can consider the model proposed in our study as a viable method to compensate for the low reliability of Zebris treadmills and similar capacitive sensor-embedded systems for VGRF measurement during walking.

## Figures and Tables

**Table 1:** The results of two-way repeated measures ANOVA for VGRFs and impulse.

Measure			Within-Subjects Effects		
			Time	Trial	Interaction
VGRF	LR	Left	F_[1.675,31.821]_ = 182.775, *p* < 0.001	F_[1,19]_ = 62.029, *p* < 0.001	F_[2.365,44.938]_ = 14.034, *p* < 0.001
		Right	F_[1.722,32.721]_ = 168.093, *p* < 0.001	F_[1,19]_ = 75.538, *p* < 0.001	F_[3.015,57.291]_ = 14.977, *p* < 0.001
	MS	Left	F_[1.512,28.730]_ = 139.023, *p* < 0.001	F_[1,19] _= 75.538, *p* < 0.001	F_[3.032,57.600]_ = 17.979, *p* < 0.001
		Right	F_[2.045,38.862]_ = 135.190, *p* < 0.001	F_[1,19]_ = 77.021, *p* < 0.001	F_[2.577,48.955]_ = 16.322, *p* < 0.001
	PO	Left	F_[1.516,28.807]_ = 93.345, *p* < 0.001	F_[1,19]_ = 51.350, *p* < 0.001	F_[2.472,46.962]_ = 3.510, *p* = 0.029
		Right	F_[1.624,30.856]_ = 98.168, *p* < 0.001	F_[1,19]_ = 32.807, *p* < 0.001	F_[2.623,49.841]_ = 5.051, *p* = 0.006
Impulse		Left	F_[1.260,23.934]_ = 120.867, *p* < 0.001	F_[1,19]_ = 52.881, *p* < 0.001	F_[1.841,34.970]_ = 18.048, *p* < 0.001
		Right	F_[1.391,26.423]_ = 134.003, *p* < 0.001	F_[1,19]_ = 56.153, *p* < 0.001	F_[2.060,39.143]_ = 21.252, *p* < 0.001

**Table 2:** The results of two-way repeated measures ANOVA performed for a sanity check.

Measure		Within-Subjects Effects		
		Time	Trial	Interaction
Stance duration	Left	F_[2.124,40.360]_ = 3.954, *p* = 0.025	F_[1,19]_ = 0.229, *p* = 0.638	F_[3.295,62.614]_ = 1.137, *p* = 0.343
	Right	F_[2.121,40.299]_ = 2.273, *p* = 0.113	F_[1,19]_ = 0.228, *p* = 0.639	F_[3.623,68.835]_ = 1.178, *p* = 0.327
Stride duration		F_[1.991,37.835]_ = 1.623, *p* = 0.211	F_[1,19]_ = 0.462, *p* = 0.505	F_[3.072,58.369]_ = 1.662, *p* = 0.184
Δ*V*_COM_		F_[5.029,95.557]_ = 0.990, *p* = 0.450	F_[1,19]_ = 1.718, *p* = 0.206	F_[4.626,87.902]_ = 0.491, *p* = 0.879

**Table 3:** The results of two-way repeated measures ANOVA for *R*^2.^

Measure	Within-Subjects Effects		
	Resolution	Trial	Interaction
Stride	F_[1.017,19.317]_ = 21.053, *p* < 0.001	F_[1,19]_ = 17.089, *p* = 0.001	F_[1.225,23.269]_ = 12.716, *p* = 0.001
Time	F_[1.100,20.906]_ = 9.526, *p* = 0.005	F_[1,19]_ = 18.839, *p* < 0.001	F_[1.476,28.038]_ = 9.769, *p* = 0.002
Distance	F_[1.035,19.661]_ = 14.530, *p* = 0.001	F_[1,19]_ = 15.773, *p* = 0.001	F_[1.238,23.526]_ =4.702, *p* = 0.033

## Data Availability

All data sets generated and/or analyzed during the current study are available from the corresponding author (J.A.) on reasonable request.

## Supplementary Materials

The followings are available online at https://www.mdpi.com/article/10.3390/s21165511/s1: Table S1: The equations of the curve-fitting model with respect to stride number and the corresponding coefficient of determination (R2) for each participant, Table S2: The equations of the curve-fitting model with respect to time and the corresponding coefficient of determination (R2) for each participant, and Table S3: The equations of the curve-fitting model with respect to distance and the corresponding coefficient of determination (R2) for each participant.
