# Supplementary material for: A Pressure-Pad-Embedded Treadmill Yields Time-Dependent Errors in Estimating Ground Reaction Force during Walking
Source: Sensors (Basel). 2021 Aug 17;21(16):5511. doi: 10.3390/s21165511 (PMC8401449; doi:10.3390/s21165511)
Supplement: Supplementary file 1 [file sensors-21-05511-s001.zip › sensors-1310284-supplementary.pdf]

Supplementary Table S1: The equations of the curve-fitting model with respect to stride number and the corresponding coefficient of determination (R^2) for each participant.

Participant No.	Trial No.	1 stride ΔI_{fit} =	5 strides ΔI_{fit} =	10 strides ΔI_{fit} =	15 strides ΔI_{fit} =	20 strides ΔI_{fit} =
**1**	1	$-311.77+253.10e^{-0.0023x}$, $R^2 = 0.972$	$-309.32+250.46e^{-0.0117x}$, $R^2 = 0.988$	$-309.71+249.29e^{-0.0234x}$, $R^2 = 0.994$	$-314.49+253.12e^{-0.0350x}$, $R^2 = 0.993$	$-314.24+251.43e^{-0.0467x}$, $R^2 = 0.994$
	2	$-324.84+131.38e^{-0.0022x}$, $R^2 = 0.914$	$-325.75+132.09e^{-0.0112x}$, $R^2 = 0.972$	$-327.87+133.67e^{-0.0222x}$, $R^2 = 0.995$	$-328.07+133.06e^{-0.0333x}$, $R^2 = 0.986$	$-328.59+132.88e^{-0.0441x}$, $R^2 = 0.989$
**2**	1	$-253.64+252.41e^{-0.0010x}$, $R^2 = 0.972$	$-235.06+251.57e^{-0.0051x}$, $R^2 = 0.991$	$-258.54+274.09e^{-0.0092x}$, $R^2 = 0.994$	$-263.00+277.89e^{-0.0135x}$, $R^2 = 0.996$	$-264.60+279.32e^{-0.0183x}$, $R^2 = 0.996$
	2	$-165.89+140.03e^{-0.0022x}$, $R^2 = 0.972$	$-169.88+143.57e^{-0.0103x}$, $R^2 = 0.990$	$-168.18+141.20e^{-0.0210x}$, $R^2 = 0.994$	$-167.68+139.91e^{-0.0316x}$, $R^2 = 0.995$	$-171.52+143.30e^{-0.0415x}$, $R^2 = 0.997$
**3**	1	$-143.49+146.89e^{-0.0022x}$, $R^2 = 0.981$	$-142.48+145.85e^{-0.0111x}$, $R^2 = 0.991$	$-142.57+145.09e^{-0.0221x}$, $R^2 = 0.992$	$-143.78+145.95e^{-0.0338x}$, $R^2 = 0.992$	$-148.23+149.41e^{-0.0431x}$, $R^2 = 0.992$
	2	$-159.78+137.24e^{-0.0024x}$, $R^2 = 0.973$	$-158.79+136.18e^{-0.0121x}$, $R^2 = 0.987$	$-159.47+136.27e^{-0.0243x}$, $R^2 = 0.989$	$-159.33+134.97e^{-0.0359x}$, $R^2 = 0.990$	$-163.72+139.09e^{-0.0478x}$, $R^2 = 0.990$
**4**	1	$-143.70+128.00e^{-0.0040x}$, $R^2 = 0.826$	$-154.40+139.46e^{-0.0168x}$, $R^2 = 0.958$	$-147.35+131.48e^{-0.0379x}$, $R^2 = 0.965$	$-154.83+137.49e^{-0.0516x}$, $R^2 = 0.977$	$-153.78+136.56e^{-0.0715x}$, $R^2 = 0.979$
	2	$-580.41+488.52e^{-0.0023x}$, $R^2 = 0.716$	$-579.63+484.42e^{-0.0037x}$, $R^2 = 0.833$	$-159.40+80.00e^{-0.0394x}$, $R^2 = 0.921$	$-163.74+82.39e^{-0.0535x}$, $R^2 = 0.923$	$-164.92+82.73e^{-0.0687x}$, $R^2 = 0.934$
**5**	1	$-244.58+163.18e^{-0.0032x}$, $R^2 = 0.985$	$-233.98+162.51e^{-0.0163x}$, $R^2 = 0.993$	$-224.53+161.60e^{-0.0324x}$, $R^2 = 0.995$	$-224.75+160.74e^{-0.0492x}$, $R^2 = 0.996$	$-226.13+161.06e^{-0.0656x}$, $R^2 = 0.996$
	2	$-281.61+132.63e^{-0.0022x}$, $R^2 = 0.960$	$-283.25+134.09e^{-0.0108x}$, $R^2 = 0.988$	$-286.05+136.21e^{-0.0211x}$, $R^2 = 0.993$	$-289.31+138.81e^{-0.0311x}$, $R^2 = 0.995$	$-289.82+138.81e^{-0.0418x}$, $R^2 = 0.995$
**6**	1	$-184.92+164.62e^{-0.0011x}$, $R^2 = 0.953$	$-206.22+185.54e^{-0.0048x}$, $R^2 = 0.981$	$-206.97+185.81e^{-0.0095x}$, $R^2 = 0.987$	$-196.71+175.25e^{-0.0153x}$, $R^2 = 0.990$	$-199.66+178.06e^{-0.0207x}$, $R^2 = 0.994$
	2	$-129.11+101.10e^{-0.0015x}$, $R^2 = 0.946$	$-132.96+104.71e^{-0.0069x}$, $R^2 = 0.977$	$-135.50+106.88e^{-0.0135x}$, $R^2 = 0.984$	$-138.40+109.34e^{-0.0195x}$, $R^2 = 0.986$	$-139.65+110.48e^{-0.0262x}$, $R^2 = 0.988$
**7**	1	$-187.10+167.38e^{-0.0024x}$, $R^2 = 0.934$	$-211.03+189.41e^{-0.0098x}$, $R^2 = 0.975$	$-212.00+189.71e^{-0.0197x}$, $R^2 = 0.985$	$-212.23+189.41e^{-0.0299x}$, $R^2 = 0.987$	$-217.05+193.38e^{-0.0391x}$, $R^2 = 0.992$

	2	$-129.11+133.99e^{-0.0022x}$,<br>$R^2 = 0.887$	$-219.27+134.20e^{-0.0112x}$,<br>$R^2 = 0.961$	$-217.75+132.32e^{-0.0231x}$,<br>$R^2 = 0.976$	$-201.25+116.15e^{-0.0443x}$,<br>$R^2 = 0.954$	$-221.25+134.53e^{-0.0439x}$,<br>$R^2 = 0.984$
**8**	1	$-262.57+224.42e^{-0.0024x}$,<br>$R^2 = 0.966$	$-261.35+223.18e^{-0.0123x}$,<br>$R^2 = 0.992$	$-261.66+222.03e^{-0.0245x}$,<br>$R^2 = 0.994$	$-260.89+219.91e^{-0.0369x}$,<br>$R^2 = 0.995$	$-259.64+217.23e^{-0.0495x}$,<br>$R^2 = 0.995$
	2	$-295.23+177.88e^{-0.0024x}$,<br>$R^2 = 0.927$	$-291.82+174.41e^{-0.0126x}$,<br>$R^2 = 0.978$	$-293.21+175.00e^{-0.0254x}$,<br>$R^2 = 0.985$	$-292.05+173.07e^{-0.0390x}$,<br>$R^2 = 0.989$	$-291.01+171.29e^{-0.0534x}$,<br>$R^2 = 0.991$
**9**	1	$-285.24+233.37e^{-0.0031x}$,<br>$R^2 = 0.964$	$-286.44+233.90e^{-0.0154x}$,<br>$R^2 = 0.988$	$-288.09+234.03e^{-0.0308x}$,<br>$R^2 = 0.992$	$-287.93+232.07e^{-0.0462x}$,<br>$R^2 = 0.995$	$-288.92+231.11e^{-0.0611x}$,<br>$R^2 = 0.995$
	2	$-297.26+138.77e^{-0.0034x}$,<br>$R^2 = 0.899$	$-297.55+139.03e^{-0.0171x}$,<br>$R^2 = 0.967$	$-297.61+137.84e^{-0.0342x}$,<br>$R^2 = 0.974$	$-298.10+136.97e^{-0.0509x}$,<br>$R^2 = 0.978$	$-297.46+135.32e^{-0.0686x}$,<br>$R^2 = 0.983$
**10**	1	$-199.41+142.21e^{-0.0015x}$,<br>$R^2 = 0.969$	$-195.44+138.45e^{-0.0079x}$,<br>$R^2 = 0.988$	$-195.63+138.06e^{-0.0158x}$,<br>$R^2 = 0.992$	$-196.82+138.86e^{-0.0238x}$,<br>$R^2 = 0.992$	$-197.54+138.78e^{-0.0310x}$,<br>$R^2 = 0.992$
	2	$-200.56+96.41e^{-0.0015x}$,<br>$R^2 = 0.934$	$-201.50+97.26e^{-0.0076x}$,<br>$R^2 = 0.969$	$-202.13+97.52e^{-0.0151x}$,<br>$R^2 = 0.976$	$-204.77+99.79e^{-0.0220x}$,<br>$R^2 = 0.985$	$-192.68+87.56e^{-0.0345x}$,<br>$R^2 = 0.985$
**11**	1	$-103.80+82.72e^{-0.0029x}$,<br>$R^2 = 0.978$	$-103.82+82.76e^{-0.0145x}$,<br>$R^2 = 0.989$	$-103.58+82.11e^{-0.0294x}$,<br>$R^2 = 0.992$	$-104.06+82.14e^{-0.0443x}$,<br>$R^2 = 0.993$	$-104.01+81.24e^{-0.0582}$,<br>$R^2 = 0.992$
	2	$-109.46+62.21e^{-0.0036x}$,<br>$R^2 = 0.961$	$-109.46+62.09e^{-0.0181x}$,<br>$R^2 = 0.980$	$-110.02+62.09e^{-0.0151x}$,<br>$R^2 = 0.985$	$-110.81+62.32e^{-0.0530x}$,<br>$R^2 = 0.987$	$-110.08+61.01e^{-0.0715x}$,<br>$R^2 = 0.988$
**12**	1	$-172.30+111.15e^{-0.0030x}$,<br>$R^2 = 0.953$	$-172.95+111.39e^{-0.0149x}$,<br>$R^2 = 0.969$	$-174.06+111.77e^{-0.0299x}$,<br>$R^2 = 0.976$	$-173.88+111.80e^{-0.0446x}$,<br>$R^2 = 0.981$	$-177.08+113.24e^{-0.0575x}$,<br>$R^2 = 0.981$
	2	$-227.62+114.64e^{-0.0013x}$,<br>$R^2 = 0.917$	$-223.77+110.97e^{-0.0070x}$,<br>$R^2 = 0.948$	$-223.92+110.70e^{-0.0140x}$,<br>$R^2 = 0.956$	$-225.14+111.45e^{-0.0207x}$,<br>$R^2 = 0.965$	$-216.75+103.22e^{-0.0316x}$,<br>$R^2 = 0.960$
**13**	1	$-245.96+224.26e^{-0.0031x}$,<br>$R^2 = 0.991$	$-245.95+223.88e^{-0.0157x}$,<br>$R^2 = 0.996$	$-248.16+224.53e^{-0.0313x}$,<br>$R^2 = 0.998$	$-248.85+223.47e^{-0.0467x}$,<br>$R^2 = 0.998$	$-249.47+222.15e^{-0.0618x}$,<br>$R^2 = 0.999$
	2	$-432.84+249.78e^{-0.0006x}$,<br>$R^2 = 0.889$	$-434.65+251.49e^{-0.0028x}$,<br>$R^2 = 0.944$	$-432.72+249.21e^{-0.0057x}$,<br>$R^2 = 0.955$	$-457.83+274.09e^{-0.0079x}$,<br>$R^2 = 0.964$	$-446.06+262.00e^{-0.0111x}$,<br>$R^2 = 0.961$
**14**	1	$-131.73+109.16e^{-0.0027x}$,<br>$R^2 = 0.957$	$-132.02+109.29e^{-0.0135x}$,<br>$R^2 = 0.981$	$-133.24+109.86e^{-0.0269x}$,<br>$R^2 = 0.985$	$-134.98+110.89e^{-0.0398x}$,<br>$R^2 = 0.986$	$-132.98+108.18e^{-0.0540x}$,<br>$R^2 = 0.987$
	2	$-169.75+108.74e^{-0.0016x}$,<br>$R^2 = 0.859$	$-170.03+108.92e^{-0.0081x}$,<br>$R^2 = 0.919$	$-170.05+108.45e^{-0.0163x}$,<br>$R^2 = 0.936$	$-186.28+123.97e^{-0.0204x}$,<br>$R^2 = 0.951$	$-171.97+109.39e^{-0.0317x}$,<br>$R^2 = 0.949$
**15**	1	$-259.10+203.79e^{-0.0017x}$,<br>$R^2 = 0.976$	$-258.98+203.46e^{-0.0083x}$,<br>$R^2 = 0.990$	$-259.70+203.26e^{-0.0166x}$,<br>$R^2 = 0.993$	$-265.85+208.95e^{-0.0244x}$,<br>$R^2 = 0.994$	$-259.80+201.66e^{-0.0331x}$,<br>$R^2 = 0.994$

	2	$-289.08+161.02e^{-0.0015x}$, $R^2 = 0.949$	$-285.80+157.73e^{-0.0078x}$, $R^2 = 0.978$	$-285.50+156.81e^{-0.0155x}$, $R^2 = 0.981$	$-290.48+161.35e^{-0.0228x}$, $R^2 = 0.983$	$-282.92+152.97e^{-0.0317x}$, $R^2 = 0.985$
**16**	1	$-236.45+201.25e^{-0.0017x}$, $R^2 = 0.976$	$-233.37+198.38e^{-0.0090x}$, $R^2 = 0.986$	$-233.33+197.39e^{-0.0180x}$, $R^2 = 0.991$	$-234.07+197.15e^{-0.0268x}$, $R^2 = 0.991$	$-234.89+197.03e^{-0.0355x}$, $R^2 = 0.995$
	2	$-291.22+194.08e^{-0.0016x}$, $R^2 = 0.968$	$-291.84+194.50e^{-0.0081x}$, $R^2 = 0.987$	$-295.53+197.50e^{-0.0161x}$, $R^2 = 0.990$	$-297.53+198.59e^{-0.0238x}$, $R^2 = 0.992$	$-296.15+196.47e^{-0.0320x}$, $R^2 = 0.992$
**17**	1	$-156.09+111.10e^{-0.0021x}$, $R^2 = 0.935$	$-151.59+106.63e^{-0.0112x}$, $R^2 = 0.977$	$-151.69+106.36e^{-0.0227x}$, $R^2 = 0.986$	$-150.74+104.69e^{-0.0341x}$, $R^2 = 0.991$	$-152.40+105.75e^{-0.0448x}$, $R^2 = 0.991$
	2	$-161.97+77.34e^{-0.0031x}$, $R^2 = 0.807$	$-160.48+75.88e^{-0.0158x}$, $R^2 = 0.939$	$-159.12+74.12e^{-0.0327x}$, $R^2 = 0.965$	$-156.90+71.85e^{-0.0528x}$, $R^2 = 0.974$	$-155.36+69.59e^{-0.0720x}$, $R^2 = 0.983$
**18**	1	$-286.66+253.12e^{-0.0021x}$, $R^2 = 0.844$	$-287.30+253.91e^{-0.0107x}$, $R^2 = 0.956$	$-295.19+259.71e^{-0.0204x}$, $R^2 = 0.979$	$-299.29+262.74e^{-0.0304x}$, $R^2 = 0.979$	$-298.84+260.28e^{-0.0399x}$, $R^2 = 0.987$
	2	$-267.26+126.48e^{-0.0030x}$, $R^2 = 0.572$	$-264.16+123.64e^{-0.0155x}$, $R^2 = 0.836$	$-266.30+124.45e^{-0.0302x}$, $R^2 = 0.893$	$-273.51+130.27e^{-0.0417x}$, $R^2 = 0.942$	$-267.48+123.39e^{-0.0590x}$, $R^2 = 0.934$
**19**	1	$-150.55+96.68e^{-0.0029x}$, $R^2 = 0.957$	$-150.61+96.17e^{-0.0141x}$, $R^2 = 0.988$	$-151.35+96.34e^{-0.0281x}$, $R^2 = 0.994$	$-151.37+96.07e^{-0.0431x}$, $R^2 = 0.988$	$-154.87+99.20e^{-0.0564x}$, $R^2 = 0.994$
	2	$-165.83+67.23e^{-0.0025x}$, $R^2 = 0.969$	$-165.00+66.47e^{-0.0128x}$, $R^2 = 0.984$	$-164.47+65.56e^{-0.0260x}$, $R^2 = 0.990$	$-166.84+67.61e^{-0.0379x}$, $R^2 = 0.992$	$-170.49+70.70e^{-0.0470x}$, $R^2 = 0.995$
**20**	1	$-149.35+84.84e^{-0.0028x}$, $R^2 = 0.889$	$-149.61+84.92e^{-0.0141x}$, $R^2 = 0.967$	$-151.41+85.98e^{-0.0273x}$, $R^2 = 0.982$	$-151.12+85.16e^{-0.0412x}$, $R^2 = 0.987$	$-155.15+88.55e^{-0.0518x}$, $R^2 = 0.989$
	2	$-167.36+70.75e^{-0.0024x}$, $R^2 = 0.738$	$-166.49+69.87e^{-0.0123x}$, $R^2 = 0.892$	$-167.38+70.31e^{-0.0243x}$, $R^2 = 0.921$	$-167.14+69.71e^{-0.0367x}$, $R^2 = 0.933$	$-167.23+69.54e^{-0.0500x}$, $R^2 = 0.951$

Supplementary Table S2: The equations of the curve-fitting model with respect to time and the corresponding coefficient of determination (R^2) for each participant.

Participant No.	Trial No.	5 seconds ΔI_{fit} =	10 seconds ΔI_{fit} =	15 seconds ΔI_{fit} =	20 seconds ΔI_{fit} =
**1**	1	$-305.68+247.24e^{-0.0100x}$, $R^2 = 0.986$	$-307.80+247.79e^{-0.0197x}$, $R^2 = 0.992$	$-308.73+247.38e^{-0.0294x}$, $R^2 = 0.994$	$-309.31+246.73e^{-0.0390x}$, $R^2 = 0.995$
	2	$-323.62+129.81e^{-0.0094x}$, $R^2 = 0.966$	$-323.34+129.12e^{-0.0198x}$, $R^2 = 0.980$	$-323.81+128.93e^{-0.0282x}$, $R^2 = 0.986$	$-323.59+128.22e^{-0.0378x}$, $R^2 = 0.991$
**2**	1	$-227.93+244.82e^{-0.0048x}$, $R^2 = 0.992$	$-228.93+245.01e^{-0.0095x}$, $R^2 = 0.995$	$-229.15+244.58e^{-0.0142x}$, $R^2 = 0.996$	$-231.66+246.47e^{-0.0187x}$, $R^2 = 0.996$
	2	$-163.75+137.85e^{-0.0099x}$, $R^2 = 0.989$	$-164.06+137.33e^{-0.0198x}$, $R^2 = 0.995$	$-164.68+137.24e^{-0.02985x}$, $R^2 = 0.996$	$-165.22+136.97e^{-0.0390x}$, $R^2 = 0.997$
**3**	1	$-144.26+147.42e^{-0.0091x}$, $R^2 = 0.988$	$-143.80+146.26e^{-0.0183x}$, $R^2 = 0.991$	$-144.20+145.98e^{-0.0274x}$, $R^2 = 0.991$	$-143.46+144.66e^{-0.0368x}$, $R^2 = 0.993$
	2	$-158.21+135.99e^{-0.0104x}$, $R^2 = 0.984$	$-158.15+135.12e^{-0.0208x}$, $R^2 = 0.987$	$-158.37+134.59e^{-0.0310x}$, $R^2 = 0.989$	$-158.73+134.21e^{-0.0412x}$, $R^2 = 0.989$
**4**	1	$-135.04+120.92e^{-0.0192x}$, $R^2 = 0.820$	$-166.27+149.27e^{-0.0229x}$, $R^2 = 0.965$	$-152.64+136.59e^{-0.0424x}$, $R^2 = 0.955$	$-158.26+140.78e^{-0.0516x}$, $R^2 = 0.970$
	2	$-541.01+447.32e^{-0.0060x}$, $R^2 = 0.738$	$-165.27+84.32e^{-0.0274x}$, $R^2 = 0.898$	$-163.02+81.83e^{-0.0433x}$, $R^2 = 0.905$	$-158.36+76.92e^{-0.0646x}$, $R^2 = 0.903$
**5**	1	$-223.76+163.60e^{-0.0127x}$, $R^2 = 0.991$	$-224.05+162.79e^{-0.0254x}$, $R^2 = 0.994$	$-224.79+162.21e^{-0.0377x}$, $R^2 = 0.996$	$-225.44+161.57e^{-0.0498x}$, $R^2 = 0.996$
	2	$-278.24+129.86e^{-0.0090x}$, $R^2 = 0.981$	$-278.91+129.75e^{-0.0178x}$, $R^2 = 0.988$	$-278.25+128.50e^{-0.0269x}$, $R^2 = 0.991$	$-280.82+129.59e^{-0.0350x}$, $R^2 = 0.993$
**6**	1	$-173.55+153.15e^{-0.0055x}$, $R^2 = 0.973$	$-179.71+158.71e^{-0.0104x}$, $R^2 = 0.985$	$-180.16+158.76e^{-0.0155x}$, $R^2 = 0.987$	$-179.01+157.17e^{-0.0208x}$, $R^2 = 0.989$
	2	$-116.75+84.97e^{-0.0004x}$, $R^2 = 0.965$	$-128.27+99.90e^{-0.0133x}$, $R^2 = 0.983$	$-127.15+98.51e^{-0.0203x}$, $R^2 = 0.985$	$-127.40+98.45e^{-0.0269x}$, $R^2 = 0.986$
**7**	1	$-210.48+189.22e^{-0.0078x}$, $R^2 = 0.969$	$-204.80+182.66e^{-0.0161x}$, $R^2 = 0.983$	$-210.34+187.52e^{-0.0233x}$, $R^2 = 0.985$	$-208.26+184.91e^{-0.0316x}$, $R^2 = 0.989$

	2	$-214.85+130.65e^{-0.0097x}$,<br>$R^2 = 0.946$	$-218.23+132.85e^{-0.0184x}$,<br>$R^2 = 0.963$	$-225.49+139.29e^{-0.0253x}$,<br>$R^2 = 0.972$	$-219.38+132.39e^{-0.0363x}$,<br>$R^2 = 0.975$
**8**	1	$-254.59+216.35e^{-0.0103x}$,<br>$R^2 = 0.990$	$-254.97+215.55e^{-0.0206x}$,<br>$R^2 = 0.994$	$-256.75+215.76e^{-0.0304x}$,<br>$R^2 = 0.995$	$-257.34+215.24e^{-0.0403x}$,<br>$R^2 = 0.996$
	2	$-291.61+173.74e^{-0.0099x}$,<br>$R^2 = 0.970$	$-302.57+183.75e^{-0.0182x}$,<br>$R^2 = 0.983$	$-291.20+171.71e^{-0.0297x}$,<br>$R^2 = 0.984$	$-291.22+170.78e^{-0.0395x}$,<br>$R^2 = 0.986$
**9**	1	$-282.82+231.39e^{-0.0130x}$,<br>$R^2 = 0.985$	$-282.48+229.50e^{-0.0261x}$,<br>$R^2 = 0.992$	$-283.63+228.98e^{-0.0388x}$,<br>$R^2 = 0.995$	$-283.85+227.58e^{-0.0517x}$,<br>$R^2 = 0.995$
	2	$-295.40+137.60e^{-0.0142x}$,<br>$R^2 = 0.949$	$-297.02+137.72e^{-0.0277x}$,<br>$R^2 = 0.970$	$-296.51+136.33e^{-0.0419x}$,<br>$R^2 = 0.972$	$-297.67+136.16e^{-0.0548x}$,<br>$R^2 = 0.979$
**10**	1	$-193.61+137.06e^{-0.0070x}$,<br>$R^2 = 0.984$	$-194.50+137.34e^{-0.0140x}$,<br>$R^2 = 0.988$	$-195.62+137.89e^{-0.0207x}$,<br>$R^2 = 0.981$	$-196.69+138.38e^{-0.0272x}$,<br>$R^2 = 0.991$
	2	$-196.03+91.93e^{-0.0071x}$,<br>$R^2 = 0.968$	$-194.80+90.44e^{-0.0145x}$,<br>$R^2 = 0.978$	$-196.39+91.64e^{-0.0213x}$,<br>$R^2 = 0.985$	$-195.66+90.61e^{-0.0287x}$,<br>$R^2 = 0.985$
**11**	1	$-102.78+81.56e^{-0.0131x}$,<br>$R^2 = 0.987$	$-102.96+81.15e^{-0.0261x}$,<br>$R^2 = 0.989$	$-102.71+80.38e^{-0.0394x}$,<br>$R^2 = 0.990$	$-103.01+80.09e^{-0.0522}$,<br>$R^2 = 0.991$
	2	$-108.24+61.37e^{-0.0168x}$,<br>$R^2 = 0.980$	$-108.53+60.94e^{-0.0331x}$,<br>$R^2 = 0.985$	$-108.85+60.60e^{-0.0491x}$,<br>$R^2 = 0.987$	$-108.75+60.04e^{-0.0656x}$,<br>$R^2 = 0.988$
**12**	1	$-171.89+110.51e^{-0.0139x}$,<br>$R^2 = 0.969$	$-175.38+112.95e^{-0.0266x}$,<br>$R^2 = 0.979$	$-171.43+108.66e^{-0.0423x}$,<br>$R^2 = 0.979$	$-172.10+108.35e^{-0.0555x}$,<br>$R^2 = 0.981$
	2	$-224.14+111.31e^{-0.0063x}$,<br>$R^2 = 0.945$	$-205.74+93.21e^{-0.0166x}$,<br>$R^2 = 0.971$	$-225.07+111.44e^{-0.0187x}$,<br>$R^2 = 0.962$	$-227.47+113.31e^{-0.0241x}$,<br>$R^2 = 0.959$
**13**	1	$-245.30+224.16e^{-0.0130x}$,<br>$R^2 = 0.995$	$-245.85+222.95e^{-0.0259x}$,<br>$R^2 = 0.997$	$-245.62+221.42e^{-0.0390x}$,<br>$R^2 = 0.997$	$-246.44+220.52e^{-0.0515x}$,<br>$R^2 = 0.998$
	2	$-422.36+239.36e^{-0.0025x}$,<br>$R^2 = 0.926$	$-435.50+252.20e^{-0.0047x}$,<br>$R^2 = 0.948$	$-443.76+260.03e^{-0.0068x}$,<br>$R^2 = 0.955$	$-447.18+263.20e^{-0.0090x}$,<br>$R^2 = 0.960$
**14**	1	$-131.29+109.16e^{-0.0122x}$,<br>$R^2 = 0.979$	$-132.38+109.28e^{-0.0240x}$,<br>$R^2 = 0.985$	$-132.02+108.25e^{-0.0362x}$,<br>$R^2 = 0.989$	$-132.39+108.02e^{-0.0480x}$,<br>$R^2 = 0.989$
	2	$-171.02+110.18e^{-0.0071x}$,<br>$R^2 = 0.903$	$-149.41+89.35e^{-0.0204x}$,<br>$R^2 = 0.961$	$-172.76+110.87e^{-0.0206x}$,<br>$R^2 = 0.939$	$-171.38+109.11e^{-0.0279x}$,<br>$R^2 = 0.947$
**15**	1	$-256.80+202.39e^{-0.0080x}$,<br>$R^2 = 0.990$	$-258.31+202.82e^{-0.0158x}$,<br>$R^2 = 0.993$	$-259.40+202.96e^{-0.0235x}$,<br>$R^2 = 0.994$	$-259.20+202.06e^{-0.0314x}$,<br>$R^2 = 0.996$

	2	$-286.76+158.53e^{-0.0072x}$, $R^2 = 0.977$	$-287.01+158.13e^{-0.0144x}$, $R^2 = 0.988$	$-284.59+155.25e^{-0.0220x}$, $R^2 = 0.986$	$-285.14+155.26e^{-0.0292x}$, $R^2 = 0.987$
**16**	1	$-231.64+196.66e^{-0.0073x}$, $R^2 = 0.978$	$-231.58+195.88e^{-0.0146x}$, $R^2 = 0.983$	$-232.65+196.07e^{-0.0217x}$, $R^2 = 0.987$	$-231.46+194.21e^{-0.0293x}$, $R^2 = 0.990$
	2	$-279.59+183.05e^{-0.0073x}$, $R^2 = 0.982$	$-279.74+182.48e^{-0.0146x}$, $R^2 = 0.988$	$-279.94+181.99e^{-0.0218x}$, $R^2 = 0.989$	$-283.57+184.56e^{-0.0282x}$, $R^2 = 0.991$
**17**	1	$-151.69+106.77e^{-0.0106x}$, $R^2 = 0.974$	$-151.63+106.18e^{-0.0213x}$, $R^2 = 0.986$	$-151.41+105.37e^{-0.0320x}$, $R^2 = 0.993$	$-151.58+105.00e^{-0.0426x}$, $R^2 = 0.994$
	2	$-162.71+77.82e^{-0.0141x}$, $R^2 = 0.934$	$-164.07+78.49e^{-0.0274x}$, $R^2 = 0.958$	$-163.87+77.80e^{-0.0414x}$, $R^2 = 0.966$	$-163.88+77.19e^{-0.0550x}$, $R^2 = 0.975$
**18**	1	$-286.07+253.92e^{-0.0095x}$, $R^2 = 0.952$	$-285.00+251.54e^{-0.0191x}$, $R^2 = 0.967$	$-287.50+252.27e^{-0.0281x}$, $R^2 = 0.981$	$-290.92+254.15e^{-0.0367x}$, $R^2 = 0.978$
	2	$-264.28+124.35e^{-0.0140x}$, $R^2 = 0.833$	$-264.75+123.98e^{-0.0279x}$, $R^2 = 0.895$	$-265.00+122.96e^{-0.0414x}$, $R^2 = 0.933$	$-266.63+123.30e^{-0.0538x}$, $R^2 = 0.957$
**19**	1	$-148.77+94.69e^{-0.0124x}$, $R^2 = 0.987$	$-148.18+93.44e^{-0.0249x}$, $R^2 = 0.991$	$-149.10+93.78e^{-0.0370x}$, $R^2 = 0.994$	$-150.07+94.26e^{-0.0488x}$, $R^2 = 0.995$
	2	$-164.93+66.85e^{-0.0109x}$, $R^2 = 0.976$	$-164.08+65.60e^{-0.0222x}$, $R^2 = 0.979$	$-165.77+66.92e^{-0.0322x}$, $R^2 = 0.987$	$-165.37+66.04e^{-0.0430x}$, $R^2 = 0.989$
**20**	1	$-149.07+84.77e^{-0.0131x}$, $R^2 = 0.960$	$-149.36+84.44e^{-0.0259x}$, $R^2 = 0.976$	$-149.20+83.73e^{-0.0390x}$, $R^2 = 0.983$	$-149.40+83.36e^{-0.0518x}$, $R^2 = 0.985$
	2	$-165.20+68.90e^{-0.0117x}$, $R^2 = 0.872$	$-165.96+68.93e^{-0.0229x}$, $R^2 = 0.907$	$-165.59+68.29e^{-0.0347x}$, $R^2 = 0.933$	$-165.24+67.54e^{-0.0466x}$, $R^2 = 0.938$

Supplementary Table S3: The equations of the curve-fitting model with respect to distance and the corresponding coefficient of determination (R^2) for each participant.

Participant No.	Trial No.	5 meters ΔI_{fit} =	10 meters ΔI_{fit} =	15 meters ΔI_{fit} =	20 meters ΔI_{fit} =
**1**	1	$-304.75+246.76e^{-0.0089x}$, $R^2 = 0.988$	$-304.40+245.26e^{-0.0178x}$, $R^2 = 0.990$	$-309.03+248.95e^{-0.0264x}$, $R^2 = 0.992$	$-307.27+245.51e^{-0.0349x}$, $R^2 = 0.993$
	2	$-325.58+132.11e^{-0.0082x}$, $R^2 = 0.964$	$-326.39+132.67e^{-0.0164x}$, $R^2 = 0.980$	$-327.84+133.40e^{-0.0242x}$, $R^2 = 0.985$	$-325.71+131.37e^{-0.0340x}$, $R^2 = 0.988$
**2**	1	$-229.31+246.38e^{-0.0041x}$, $R^2 = 0.990$	$-242.03+258.52e^{-0.0078x}$, $R^2 = 0.993$	$-231.09+247.00e^{-0.0122x}$, $R^2 = 0.994$	$-245.84+261.18e^{-0.0153x}$, $R^2 = 0.996$
	2	$-168.30+142.38e^{-0.0082x}$, $R^2 = 0.990$	$-169.12+142.77e^{-0.0164x}$, $R^2 = 0.994$	$-168.44+141.30e^{-0.0245x}$, $R^2 = 0.996$	$-169.78+142.10e^{-0.0324x}$, $R^2 = 0.996$
**3**	1	$-143.12+146.48e^{-0.0108x}$, $R^2 = 0.993$	$-143.91+146.63e^{-0.0218x}$, $R^2 = 0.992$	$-143.72+145.42e^{-0.0323x}$, $R^2 = 0.995$	$-151.18+152.52e^{-0.0418x}$, $R^2 = 0.994$
	2	$-158.01+135.97e^{-0.0122x}$, $R^2 = 0.990$	$-158.99+136.28e^{-0.0245x}$, $R^2 = 0.992$	$-158.78+134.88e^{-0.0363x}$, $R^2 = 0.993$	$-163.45+139.22e^{-0.0481x}$, $R^2 = 0.993$
**4**	1	$-143.51+129.30e^{-0.0166x}$, $R^2 = 0.889$	$-150.16+136.38e^{-0.0306x}$, $R^2 = 0.968$	$-148.64+132.85e^{-0.0458x}$, $R^2 = 0.976$	$-152.84+136.59e^{-0.0584x}$, $R^2 = 0.980$
	2	$-568.05+476.58e^{-0.0059x}$, $R^2 = 0.881$	$-160.48+81.13e^{-0.0341x}$, $R^2 = 0.905$	$-159.01+78.65e^{-0.0498x}$, $R^2 = 0.937$	$-163.11+81.71e^{-0.0631x}$, $R^2 = 0.932$
**5**	1	$-224.98+165.00e^{-0.0131x}$, $R^2 = 0.993$	$-224.49+163.82e^{-0.0268x}$, $R^2 = 0.995$	$-225.17+163.15e^{-0.0395x}$, $R^2 = 0.996$	$-226.11+162.90e^{-0.0527x}$, $R^2 = 0.997$
	2	$-277.42+129.27e^{-0.0094x}$, $R^2 = 0.986$	$-279.11+130.45e^{-0.0187x}$, $R^2 = 0.991$	$-286.66+136.66e^{-0.0259x}$, $R^2 = 0.995$	$-289.42+138.89e^{-0.0338x}$, $R^2 = 0.996$
**6**	1	$-201.49+180.64e^{-0.0039x}$, $R^2 = 0.982$	$-197.09+175.77e^{-0.0081x}$, $R^2 = 0.986$	$-207.59+186.14e^{-0.0116x}$, $R^2 = 0.991$	$-198.68+176.78e^{-0.0160x}$, $R^2 = 0.991$
	2	$-404.48+370.27e^{-0.0011x}$, $R^2 = 0.960$	$-123.74+95.79e^{-0.0126x}$, $R^2 = 0.981$	$-128.10+100.15e^{-0.0182x}$, $R^2 = 0.989$	$-124.65+95.17e^{-0.0249x}$, $R^2 = 0.987$
**7**	1	$-206.66+185.78e^{-0.0076x}$, $R^2 = 0.969$	$-203.82+182.90e^{-0.0160x}$, $R^2 = 0.981$	$-192.99+172.23e^{-0.0263x}$, $R^2 = 0.989$	$-193.65+172.55e^{-0.0359x}$, $R^2 = 0.986$

	2	$-216.27+132.23e^{-0.0092x}$,<br>$R^2 = 0.949$	$-218.49+135.28e^{-0.0187x}$,<br>$R^2 = 0.966$	$-218.45+133.12e^{-0.0284x}$,<br>$R^2 = 0.978$	$-210.79+125.72e^{-0.0419x}$,<br>$R^2 = 0.979$
**8**	1	$-251.36+213.87e^{-0.0097x}$,<br>$R^2 = 0.989$	$-255.97+216.00e^{-0.0186x}$,<br>$R^2 = 0.991$	$-259.06+218.47e^{-0.0274x}$,<br>$R^2 = 0.993$	$-254.91+215.09e^{-0.0390x}$,<br>$R^2 = 0.992$
	2	$-294.08+176.06e^{-0.0088x}$,<br>$R^2 = 0.969$	$-298.72+179.91e^{-0.0171x}$,<br>$R^2 = 0.978$	$-289.54+170.23e^{-0.0277x}$,<br>$R^2 = 0.982$	$-295.95+175.66e^{-0.0355x}$,<br>$R^2 = 0.984$
**9**	1	$-284.17+232.57e^{-0.0132x}$,<br>$R^2 = 0.986$	$-288.51+235.06e^{-0.0261x}$,<br>$R^2 = 0.994$	$-288.55+233.80e^{-0.0394x}$,<br>$R^2 = 0.995$	$-292.09+234.98e^{-0.0516x}$,<br>$R^2 = 0.996$
	2	$-298.53+140.62e^{-0.0140x}$,<br>$R^2 = 0.959$	$-297.05+138.07e^{-0.0294x}$,<br>$R^2 = 0.976$	$-296.01+136.71e^{-0.0453x}$,<br>$R^2 = 0.981$	$-301.45+139.08e^{-0.0569x}$,<br>$R^2 = 0.987$
**10**	1	$-190.73+134.19e^{-0.0070x}$,<br>$R^2 = 0.989$	$-190.86+133.85e^{-0.0141x}$,<br>$R^2 = 0.992$	$-197.97+140.09e^{-0.0194x}$,<br>$R^2 = 0.993$	$-199.32+141.22e^{-0.0263x}$,<br>$R^2 = 0.996$
	2	$-198.05+93.99e^{-0.0070x}$,<br>$R^2 = 0.979$	$-198.95+94.66e^{-0.0133x}$,<br>$R^2 = 0.987$	$-200.46+95.87e^{-0.0193x}$,<br>$R^2 = 0.991$	$-195.24+90.45e^{-0.0284x}$,<br>$R^2 = 0.992$
**11**	1	$-103.68+82.57e^{-0.0115x}$,<br>$R^2 = 0.989$	$-102.54+81.06e^{-0.0236x}$,<br>$R^2 = 0.990$	$-104.36+82.34e^{-0.0346x}$,<br>$R^2 = 0.994$	$-102.65+80.10e^{-0.0469}$,<br>$R^2 = 0.992$
	2	$-109.67+62.79e^{-0.0143x}$,<br>$R^2 = 0.982$	$-109.93+62.32e^{-0.0286x}$,<br>$R^2 = 0.984$	$-110.29+62.54e^{-0.0430x}$,<br>$R^2 = 0.991$	$-110.37+61.94e^{-0.0580x}$,<br>$R^2 = 0.988$
**12**	1	$-172.64+111.48e^{-0.0129x}$,<br>$R^2 = 0.972$	$-173.90+111.79e^{-0.0252x}$,<br>$R^2 = 0.975$	$-175.37+111.59e^{-0.0366x}$,<br>$R^2 = 0.979$	$-173.49+109.70e^{-0.0507x}$,<br>$R^2 = 0.980$
	2	$-220.50+107.83e^{-0.0061x}$,<br>$R^2 = 0.950$	$-214.89+102.52e^{-0.0134x}$,<br>$R^2 = 0.957$	$-217.46+104.01e^{-0.0193x}$,<br>$R^2 = 0.963$	$-217.58+103.68e^{-0.0253x}$,<br>$R^2 = 0.959$
**13**	1	$-246.98+226.23e^{-0.0130x}$,<br>$R^2 = 0.996$	$-246.87+225.20e^{-0.0265x}$,<br>$R^2 = 0.997$	$-252.08+228.53e^{-0.0385x}$,<br>$R^2 = 0.998$	$-252.23+227.71e^{-0.0519x}$,<br>$R^2 = 0.998$
	2	$-420.69+237.88e^{-0.0026x}$,<br>$R^2 = 0.941$	$-417.23+234.27e^{-0.0053x}$,<br>$R^2 = 0.948$	$-439.13+255.56e^{-0.0073x}$,<br>$R^2 = 0.957$	$-410.46+226.37e^{-0.0046x}$,<br>$R^2 = 0.961$
**14**	1	$-135.79+113.12e^{-0.0101x}$,<br>$R^2 = 0.977$	$-137.61+114.13e^{-0.0199x}$,<br>$R^2 = 0.984$	$-136.71+113.24e^{-0.0309x}$,<br>$R^2 = 0.987$	$-137.36+112.55e^{-0.0398x}$,<br>$R^2 = 0.988$
	2	$-161.81+101.56e^{-0.0072x}$,<br>$R^2 = 0.908$	$-176.62+115.19e^{-0.0120x}$,<br>$R^2 = 0.934$	$-180.84+118.75e^{-0.0170x}$,<br>$R^2 = 0.945$	$-165.19+102.85e^{-0.0280x}$,<br>$R^2 = 0.949$
**15**	1	$-263.15+208.67e^{-0.0073x}$,<br>$R^2 = 0.991$	$-269.24+213.76e^{-0.0141x}$,<br>$R^2 = 0.993$	$-272.09+215.38e^{-0.0206x}$,<br>$R^2 = 0.996$	$-265.06+209.28e^{-0.0299x}$,<br>$R^2 = 0.995$

	2	$-277.85+150.52e^{-0.0076x}$, $R^2 = 0.975$	$-276.92+149.57e^{-0.0154x}$, $R^2 = 0.983$	$-290.87+162.38e^{-0.0207x}$, $R^2 = 0.986$	$-283.46+154.95e^{-0.0297x}$, $R^2 = 0.984$
**16**	1	$-231.29+197.04e^{-0.0071x}$, $R^2 = 0.980$	$-226.50+191.49e^{-0.0148x}$, $R^2 = 0.984$	$-220.39+184.73e^{-0.0239x}$, $R^2 = 0.992$	$-216.85+181.23e^{-0.0331x}$, $R^2 = 0.994$
	2	$-285.90+188.51e^{-0.0066x}$, $R^2 = 0.982$	$-289.35+191.23e^{-0.0130x}$, $R^2 = 0.985$	$-290.93+191.83e^{-0.0191x}$, $R^2 = 0.990$	$-303.52+204.49e^{-0.0243x}$, $R^2 = 0.989$
**17**	1	$-148.78+104.78e^{-0.0105x}$, $R^2 = 0.984$	$-147.34+103.47e^{-0.0217x}$, $R^2 = 0.990$	$-146.58+101.90e^{-0.0326x}$, $R^2 = 0.995$	$-145.69+101.16e^{-0.0456x}$, $R^2 = 0.994$
	2	$-160.30+76.82e^{-0.0141x}$, $R^2 = 0.963$	$-163.89+79.30e^{-0.0252x}$, $R^2 = 0.985$	$-167.10+81.42e^{-0.0356x}$, $R^2 = 0.990$	$-165.42+80.02e^{-0.0494x}$, $R^2 = 0.995$
**18**	1	$-281.04+251.13e^{-0.0087x}$, $R^2 = 0.937$	$-302.25+271.76e^{-0.0153x}$, $R^2 = 0.964$	$-297.49+268.23e^{-0.0236x}$, $R^2 = 0.972$	$-299.74+268.43e^{-0.0315x}$, $R^2 = 0.976$
	2	$-259.03+121.89e^{-0.0131x}$, $R^2 = 0.795$	$-260.63+1242.71e^{-0.0241x}$, $R^2 = 0.874$	$-264.33+125.03e^{-0.0347x}$, $R^2 = 0.923$	$-282.21+143.39e^{-0.0370x}$, $R^2 = 0.941$
**19**	1	$-150.14+95.90e^{-0.0121x}$, $R^2 = 0.995$	$-149.64+95.25e^{-0.0248x}$, $R^2 = 0.997$	$-151.90+96.62e^{-0.0359x}$, $R^2 = 0.998$	$-150.46+94.91e^{-0.0496x}$, $R^2 = 0.998$
	2	$-164.44+66.41e^{-0.0111x}$, $R^2 = 0.990$	$-162.54+64.40e^{-0.0237x}$, $R^2 = 0.993$	$-163.04+64.63e^{-0.0358x}$, $R^2 = 0.995$	$-167.04+67.84e^{-0.0434x}$, $R^2 = 0.994$
**20**	1	$-149.23+84.98e^{-0.0121x}$, $R^2 = 0.984$	$-149.81+85.21e^{-0.0241x}$, $R^2 = 0.990$	$-148.65+84.37e^{-0.0375x}$, $R^2 = 0.994$	$-150.82+85.10e^{-0.0484x}$, $R^2 = 0.994$
	2	$-167.33+70.24e^{-0.0100x}$, $R^2 = 0.940$	$-168.96+72.29e^{-0.0197x}$, $R^2 = 0.956$	$-166.62+69.35e^{-0.0308x}$, $R^2 = 0.973$	$-167.00+70.16e^{-0.0424x}$, $R^2 = 0.970$
